# Quantification and clustering of phenotypic screening data using time-series analysis for chemotherapy of schistosomiasis

**DOI:** 10.1186/1471-2164-13-S1-S4

**Published:** 2012-01-17

**Authors:** Hyokyeong Lee, Asher Moody-Davis, Utsab Saha, Brian M Suzuki, Daniel Asarnow, Steven Chen, Michelle Arkin, Conor R Caffrey, Rahul Singh

**Affiliations:** 1 Department of Computer Science, San Francisco State University, San Francisco, CA 94132, USA; 2 Open University Program, San Francisco State University, San Francisco, CA 94132, USA; 3 Sandler Center for Drug Discovery, University of California, San Francisco, CA 94158, USA; 4 Department of Chemistry and Biochemistry, San Francisco State University, San Francisco, CA 94132, USA; 5 Small Molecule Discovery Center, University of California, San Francisco, CA 94158, USA; 6 Department of Pathology, University of California, San Francisco, CA 94158, USA

## Abstract

**Background:**

Neglected tropical diseases, especially those caused by helminths, constitute some of the most common infections of the world's poorest people. Development of techniques for automated, high-throughput drug screening against these diseases, especially in whole-organism settings, constitutes one of the great challenges of modern drug discovery.

**Method:**

We present a method for enabling high-throughput phenotypic drug screening against diseases caused by helminths with a focus on schistosomiasis. The proposed method allows for a quantitative analysis of the systemic impact of a drug molecule on the pathogen as exhibited by the complex continuum of its phenotypic responses. This method consists of two key parts: first, biological image analysis is employed to automatically monitor and quantify shape-, appearance-, and motion-based phenotypes of the parasites. Next, we represent these phenotypes as time-series and show how to compare, cluster, and quantitatively reason about them using techniques of time-series analysis.

**Results:**

We present results on a number of algorithmic issues pertinent to the time-series representation of phenotypes. These include results on appropriate representation of phenotypic time-series, analysis of different time-series similarity measures for comparing phenotypic responses over time, and techniques for clustering such responses by similarity. Finally, we show how these algorithmic techniques can be used for quantifying the complex continuum of phenotypic responses of parasites. An important corollary is the ability of our method to recognize and rigorously group parasites based on the variability of their phenotypic response to different drugs.

**Conclusions:**

The methods and results presented in this paper enable automatic and quantitative scoring of high-throughput phenotypic screens focused on helmintic diseases. Furthermore, these methods allow us to analyze and stratify parasites based on their phenotypic response to drugs. Together, these advancements represent a significant breakthrough for the process of drug discovery against schistosomiasis in particular and can be extended to other helmintic diseases which together afflict a large part of humankind.

## Background

Neglected tropical diseases (NTDs) constitute the most common infections of the world's poorest people. This class of diseases encompasses a number of infection categories including helminth infections (schistosomiasis, lymphatic filariasis, onchocerciasis), protozoan infections (leishmaniasis, Chagas' disease, African trypanosomiasis), bacterial infections (cholera, leprosy, bovine tuberculosis), viral infections (dengue fever, rabies, yellow fever), fungal infections (*Mycetoma*, paracoccidiomycosis), and ectoparasitic infections (scabies, myiasis). Various studies have indicated NTDs to be the prime factors behind depriving the affected populations, especially women and children, of their health and economic potential [1,2]. This paper proposes a novel algorithmic approach to drug screening against schistosomiasis based on time-series analysis of phenotypes exhibited by parasites in response to different drug molecules. These phenotypes are themselves determined by automatically analyzing images from high-throughput screens.

Schistosomiasis in humans is caused by three major species of trematodes, *Schistosoma mansoni*, *Schistosoma haematobium *and *Schistosoma japonicum*. Affecting over 200 million people (with 20 million suffering severe effects) and placing over 800 million people at risk, schistosomiasis ranks second only behind malaria in terms of socio-economic and public health impact in developing countries. The disease starts as an inflammatory response to the eggs of the parasites and leads to fibrotic granulomatous, causing portal vein hypertension or occlusion (intestinal schistosomiasis caused by *S. mansoni*) or hydronephrosis and squamous bladder cancer (urinary schistosomiasis caused by *S. haematobium*). Greatest infection intensities occur among children and adolescents, and the disease is known to undermine social and economic development in areas of high transmission [3-5]. Over the last 30 years, treatment and control of schistosomiasis have come to rely on a single chemotherapeutic called praziquantel (PZQ). PZQ is given orally as a single-dose, has few side effects, and has bioactivity against the aforementioned three major species infecting humans. In spite of the advantages of PZQ, the reliance on a single drug to treat over 200 million people raises several serious problems. *First*, the emergence of drug resistance and possible drug failure is a major concern [3,6-8]. Indeed, increased parasite tolerance for the drug has been selected for in rodent hosts [9] and has been reported clinically in Egypt and sub-Saharan Africa [10-13]. *Second*, PZQ has important deficiencies in its therapeutic profile; cure rates vary usually between 60 - 90% [6-8]. Furthermore, the drug acts preferentially against the adult parasite, being markedly less effective (by 60 - 100%) against the juvenile schistosomula between 21 and 28 days old [14-16]. This decreased efficacy necessitates the re-treatment of individuals harboring previously juvenile parasites and potentiates the risk for resistance by exposing (partially) refractory parasites to sub-curative doses [7,17]. The World Health Organization (WHO) *has therefore declared schistosomiasis a disease for which new therapies are urgently needed *[18].

Modern drug discovery conventionally begins by identifying a molecular target (typically a protein or an enzyme) associated with a disease. Next, a large number of putative drug molecules are screened for activity against the target in in-vitro high-throughput screens (HTS) to identify "hits" which are passed onto later stages of the drug discovery pipeline for chemical optimization, optimization of the drug pharmacokinetics and pharmacodynamics, and ultimately clinical trials. The initial screening stage can typically involve a very large number of molecules (hundreds of thousands to millions), since even small variations in structure can significantly influence activity against the target. Given this context, we note that HTS platforms for *Schistosoma*, using purified protein targets are almost unknown, with the very recent exception of a target-based campaign to identify inhibitors of *S. mansoni *thioredoxin/glutathione reductase (TGR) [19]. Traditionally, researchers have tried to ameliorate this impediment by directly screening against the pathogen, in what can be termed as *whole-organism screens*. In such screens, typically a small number of molecules are tested by exposing the pathogen to them *in vitro *and the effects of the drug are captured using manual observations. Against schistosomiasis, examples of such screens include [20-24] and have led to the discovery of drugs such as praziquantel and artemisinin [6].

**Figure 1:**
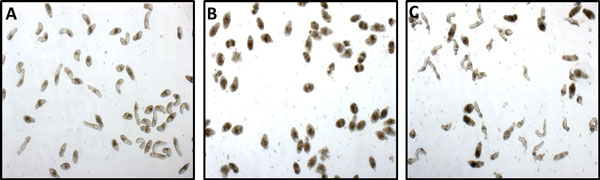
**Examples of phenotypes exhibited by the schistosomula;** (A) control (B) when exposed to the drug Lovastatin, and (C) when exposed to the drug Praziquantel (PZQ).

The whole-organism screening approach differs from the conventional HTS-based strategy. HTS is built around the use of in-vitro single enzyme activity-based screens, single read-out cell-based assays, and involves very large number of molecules which are tested in parallel using 96-, 384- or 1536-well plates. The distinctions of whole-organism screening from HTS, lead to both advantages and disadvantages. A crucial advantage is that the effect of a drug molecule can be studied in terms of the cumulative systemic effects it introduces in the parasite, rather than just in terms of how it interacts with a specific protein or enzyme in isolation. That is, the effects of the drug on the totality of targets and pathways can be explored in whole-organism screens. This can be expected to reduce the possibility of late-stage attrition of hits found through such screens. On the other hand, whole organism screens tend to be low throughput and are not easily extendable to HTS settings. This constrains, both in terms of diversity and density, the exploration of the chemical space during lead-identification. Finally, as multi-cellular organisms, schistosomes display multiple and changing phenotypes in response to how compounds interfere with their normal bio-chemical functioning. (see Figure 1). Capturing and quantifying the activity of a drug in terms of such rich and time-varying responses involves overcoming challenges of data processing, analysis, and modeling that are significantly more complex than those encountered in single end-point assays common to biochemical target-based or cell-based screens [25].

### Problem characteristics and proposed solutions

An important long-term goal in the development of drugs against NTDs in general and schistosomiasis in particular, involves the development of high-throughput whole-organism screening methods. In the following, we enumerate some of the key challenges towards solving this problem and summarize the contribution of this paper towards addressing each of the challenges:

*1. Facilitating automated high-throughput data capture and quantitative phenotype representation*: The use of single end-point measurement of 'live or death' (e.g., ED_50 _value) is over-simplistic when dealing with a multi-cellular and complex macro-parasites that can manifest a variety of temporally varying phenotypes. The need to screen compound libraries based on quantification of complex phenotypic responses of pathogens is also underlined by the fact that a drug may not necessarily lead to immediate death yet nonetheless perturb the parasite's ability to survive, e.g., through disruption of the larval migration program, tegumental perturbations releasing antigens targeted by the immune system, or the ability of adult worms to maintain position within the predilection site. As an example, the drug PZQ produces both tetanic paralysis of the musculature, resulting in loss of position as well as tegumental damage, and the exposure of surface proteins that then contribute to an immune system-mediated attack on the parasites. We propose an image analysis-based approach for automatic segmentation and tracking of parasites and computation of descriptors that capture phenotypic responses in terms of changes in parasite shape, appearance, and motion. These descriptors are represented as time-series and provide a multi-dimensional time-varying representation of parasite phenotypes.

*2. Analysis of phenotypes over time: *We propose the use of time-series clustering to compare, differentiate and analyze the phenotypic response of parasites to different drugs. Given the high-dimensional nature of the data and its sheer quantity, we investigate both numeric and symbolic representations of phenotypic time-series. We also investigate the applicability of different distance measures for comparing phenotypic responses.

*3. Dealing with variability of phenotypic responses*: A challenge in analyzing data from whole-organism screens involving schistosomes is that they cannot yet be cloned. Furthermore, schistosome development in culture is asynchronous. Consequently, the response to a drug can show much greater variability than what is seen in molecular-target based or cell-based screening due to factors such as genetic variability, lack of synchronization, gender, stochastic noise, epigentics, and possibly the differentiated levels of resistance amongst individuals. Using the formalism of time-series clustering, we show how this issue can be rigorously analyzed and examine the question of possible stratification of the phenotypic responses of different parasites that are exposed to the same drug molecule.

*4. Identification of representative phenotypic models: *Given the inherent genotypic-phenotypic variability of parasites, identification of representative phenotypic models can be valuable for understanding the core trends in the data. From a statistical perspective, a representative time-series can also be perceived as a description of the central tendencies in the data. We cast the problem of determining phenotypic models as that of finding representative time series for each phenotype cluster in the data. We present different algorithmic methods to address this question, and discuss case studies that show its usefulness for information summarization and presentation.

### Distinctions from prior research

The use of quantitative phenotyping in biology and drug discovery has occurred along two directions. The first of these involves the study of phenotypic variations in model organisms such as *C. elegans*, as a function of gene-knockouts or changes in environmental conditions. The second direction involves dug screening based on cytological profiling. The distinctions of our problem formulations, methods, and results from research in both these directions are significant. Compared to the *C. elegans *mutants [26-30], the morphology and appearance of schistosomula are more complex and undergo a greater variety of changes when exposed to different compounds as clones of genotypically identical schistosomes do not exist. Furthermore, our input data consists of images and video of multiple parasites residing in multi-well plates used in HTS. This leads to imaging conditions very different than those used for *C. elegans *(which typically involve large Petri-dishes with only few worms per dish). Our problem formulation shares the goal of lead identification with cytological screening [31-33]. However, our challenges are more acute and complex: the phenotypic response of (genetically diverse) parasites tends to be both more nuanced as well as more diverse than what is observed in cell-based screens. Furthermore, analysis of cellular phenotypes can use simplified models of cell shape. As we shall discuss in the next section, such models cannot be easily obtained for *Schistosoma*.

The investigations and results presented in this paper extend the framework proposed by Singh et al. for automated phenotypic screening [34]. The method proposed in [34] used mean-shift-based segmentation & tracking of parasites. In it, phenotypes were classified using classification and regression trees (CART). The method presented in this paper differs from [34] in terms of the algorithmic approach used for parasite segmentation and tracking. Further, in this paper, we use the entire response of the parasite (represented as a time-series) for phenotype analysis. In [34] on the other hand, CART-based classification was performed on each individual frame of the video. Finally, to the best of our knowledge, neither time-series analysis, nor the specific problems investigated by us in terms of representation and analysis of phenotypic variability have been considered either for investigation of *C. elegans *phenotypes or for cytological profiling.

## Method

### Parasite identification by image segmentation

In contrast to cellular segmentation, a topic that has received considerable attention in bio-image analysis, the problem of segmenting schistosomula in drug screens presents certain specific challenges. *First*, unlike cells, a geometric or appearance-based model of parasite shape cannot be assumed *a priori*. This is due to a number of factors including the fact that the movement of the schistosomula is based on elongation and contraction of their musculature. Therefore, the shape of the parasite's body can change considerably within a single movement cycle. Furthermore, drug action can distort the parasite body and appearance in unique manners (as can be seen in Figure 1). *Second*, parasites contain visible "inner" anatomical structures that complicate segmentation by creating edges that do not correspond to the boundaries of the body. *Third*, parasites often touch each other in varying configurations. This often complicates identification of individuals. *Finally*, observation periods can be long (days) and debris in the background can accumulate leading to an increase in the false positives. Our approach to parasite segmentation builds on prior research on segmentation of cells and parasites in biological images [34-36]. The basic idea involves a multi-step process, where the first step distinguishes between the foreground (corresponding to the parasites) and the background. In the second step, the foreground objects are analyzed and filtered to remove false positives. In the final step, a series of morphological operators are applied to separate individual parasites. In the following, we describe each of these steps in detail.

In order to distinguish the background from the parasite, we modify and extend the region-based voting approach from [36]. First a low-pass filter is applied to the image to remove noise and smooth the image. Next an adjustable threshold γ which is described later in this section is subtracted from the image. The result is asymptotically bounded using a sigmoid function to restrict values to between +1 and -1, resulting in pixel values below the threshold γ being skewed towards the background because they have a higher weight. Subsequently, in a manner similar to [36], a region-based distributing function (RDF) *R_1 _*defined in Eq.(1), is applied to the image. The purpose of this function is to rapidly identify the background by assigning a higher weight to background pixels. The functional form of the RDF used by us differs from the one proposed in [36] in two ways: first, Eq.(1) contains only a single parameter γ and second, this parameter is interpreted in our approach as a threshold on the intensity difference between the background and foreground.

(1) $$R_{1}\left(n\right)=-sig\left(\left(f⊗h\right)\left(n\right)-\gamma \right)$$

(2) $$\gamma =\min\left\{\left(I_{\max}-I_{\min}\right)∕2,\;\lambda \right\}$$

In Eq.(1), *f *denotes the image, *h *the low-pass filter, ⊗ the convolution operator and *sig(.) *the sigmoid function. The starting threshold *γ *is determined as per Eq.(2), where *I_max _*and *I_min _*denote the highest and lowest intensity values in the image. The value of the cutoff *λ *in Eq.(2) was empirically established to be 50 based on an intensity range of [0, 255]. The region-based distributing function is iteratively applied with a decreasing threshold. As the threshold γ decreases, that is, as the difference in the foreground and background intensities becomes smaller, the segmented results increasingly begin to include regions with intensity values closer to the background intensity. Initially, this increases the number of true positives (parasites) and progressively false positives occur. In the limit, the number of foreground objects decreases to one (as the entire image is treated as the foreground). Thus, a graph of the number of foreground objects has a left-shifted single mode. We found that tolerating a certain amount of false positives led to segmentations with the largest number of actual parasites correctly distinguished. Consequently, the threshold value which leads to the greatest increase in the number of segmented regions in the image is used to define γ. The false positives generated as a consequence of the oversegmentation tend to be small regions (of less than 200 pixels) representing debris from the background and are removed using region-size-based filtering. An intensity-based filter is subsequently applied as part of the filtering process. In it, the average intensity of each foreground region is compared to the overall average intensity of all the foreground regions. Regions with values outside of one and half times the standard deviation are considered background and removed.

**Figure 2:**
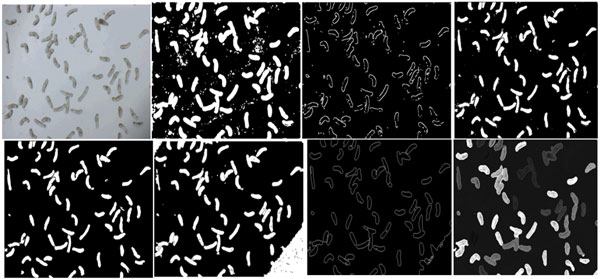
**Illustration of the segmentation results and comparison with other methods. Top row **from left-to-right: The original image (note that the bottom right region has a shadow), results of the region-based distributing function showing oversegmentation, relevant edges, and the image after filtering of debris and small regions. **Bottom row **from left-to-right: Final results with the proposed method after closing and filling holes in regions and separation of touching parasites, results obtained by mean-shift segmentation [7], results of JSEG segmentation [9], and results from the active-mask segmentation method [35].

In the final step of the segmentation, morphological processing is employed to separate touching parasites. It begins by detecting the edges of the original image. For this, the Canny edge operator [37] is used and the detected edges are subtracted from the foreground image. A dilation-erosion step is next applied to remove internal gaps that might have occurred due to edge subtraction. Next, *relevant edge pixels *are found and subtracted from the foreground. These pixels correspond to edges that separate connected components. In terms of image connectivity this means that every such edge pixel in the labeled image must have at least two different labels in its 8 neighbors. The removal of relevant edges results in separation of two different regions that are touching. The results from different stages of this method are shown in Figure 2 (top row). The first image in Figure 2 (bottom row) shows the final segmentation result. Other images in the bottom row illustrate the complexity of the segmentation problem by presenting results obtained on this image using three well known segmentation techniques: mean-shift [38], JSEG [39], and active-mask [36]. As can be seen, all of these methods face difficulty in distinguishing touching parasites.

### Parasite tracking

**Figure 3:**
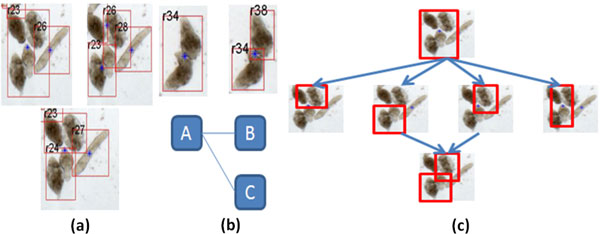
(a) Parasites can be located in close proximity to each other in manners that lead to segmentation errors. (b) Bipartite graph describing the splitting of a blob (containing two parasites) into two blobs containing a single parasite each. (c) Group of four parasites, erroneously assigned to a single blob after segmentation. Analysis of the various combinations of intensity-boxes leads to the recovery of one of the four parasites from this blob.

The ability to analyze time-varying phenotypic response of parasites requires tracking each parasite across the entire video sequence. Given an initial segmentation, for each parasite, this involves establishing a correspondence between its positions in successive frames. Once the parasites are tracked across the video, their appearance, shape, and motion can be described quantitatively. In designing a tracking system for the parasites in HTS, the following challenges have to be addressed:

1. Robust handling of the erroneous or ambiguous segmentation of the parasites. Specifically, due to their tendency to mingle, errors in segmentation can result in clusters of parasites merging and splitting in a variety of ambiguous combinations (see Figure 3(a)). A closely related problem involves dealing with cases, where one parasite is incorrectly segmented into two regions.

2. Precision in defining individual parasites, so that the phenotypes can be accurately measured over the entire duration of observation. This is especially important since we plan to use the entire phenotypic response of the parasite in our analysis.

3. Accounting for the unique motion characteristics of the parasites; unlike many problems in vision-based tracking where the object being tracked moves rapidly, schistosomula can exhibit significant movement due to twists and turns of their bodies, without appreciable translation of their body positions.

We design our tracking approach to consist of three conceptual levels: the *segmentation level*, the *blob level*, and the *parasite level*. The segmentation method described in the previous section constitutes the segmentation level. The blob level is based on a graph-theoretic approach to track blobs proposed in [40]. This method offers a data-driven model for representing the merging and splitting behavior of regions. However, this approach assumes that objects can be reliably separated from the background based on motion and does not deal with issues arising out of segmentation errors that can occur in cluttered settings, such as when a single parasite is erroneously split across multiple regions or when multiple parasites are too close to be separated. We propose a novel approach to deal with these challenges in the parasite level. In the following, we describe the blob and parasite levels.

In the blob level, each distinct foreground region (putatively representing a parasite) is represented using its bounding box in the *xy*-coordinate (image) plane. In the following, we call each such region a *blob*. Next, an undirected bipartite graph ***G_i_***(*V_i_,E_i_*) is used to model associations between blobs in consecutive frames such that *V_i_*=*B_i_*∪*B_i-1_*, where *B_i _*and *B_i-1 _*are sets of vertices corresponding to blobs in frames *i *and *i-1*. Specifically, an association between a blob *u *in frame *i-1 *and a blob *v *in frame *i *is represented as an edge (*u, v*) in ***G_i_***. In this graph, two blobs that share an edge are called neighbors and the set of neighbors of a vertex (blob) *u *is denoted by *N_i_*(*u*). Further, the number of associations of a blob *u *is described by its degree, *deg*(*u*). Figure 3(b)) illustrates an example graph of blob associations. The advantage of the blobs model is that it allows for the definition of many-to-many relationship between blobs throughout the video, such that blobs can merge, split, appear, or vanish depending on the information obtained from the segmentation level. Following [40], two constraints are used to reduce the set of edges in ***G_i_***: the l*ocality constraint*, requiring that two blobs be sufficiently close to each other in order to be considered as neighbors and the p*arent structure constraint *requiring that a blob not merge and split simultaneously. Finally, the correspondence between blobs is determined by enumerating the set of all edges between blobs in frame *i-1 *and those in frame *i *and ranking the resulting graphs to choose the most viable correspondence. To design a cost function for ranking graphs, two disjoint sets, called parents *P_i _*and descendants *D_i_*, are defined Eq(3)-Eq(5). Of these, Eq.(3) and Eq.(4) are straightforward. Eq.(5) captures the relationship that the parent set can be computed by taking all vertices of degree greater than one, all vertices of degree zero, and all vertices of degree one which are only in blob *B_i-1_*.

(3) $$V_{i}=P_{i}\cup D_{i}$$

(4) $$D_{i}=\cup N_{i}\left(u\right),\;u\in P_{i}$$

(5) $$P_{i}=\;u|\deg\left(u\right)>1\cup \;v|\deg\left(v\right)=0\cup w|\deg\left(w\right)=1\wedge w\in B_{i-1}$$

The cost function used to rank the graphs is shown in Eq.(6). In this equation, *A(u) *denotes the area of blob *u*, the summation corresponds to the total area occupied by the neighboring blobs of blob *u*, and *P_i _*denotes the parent set. This function penalizes graphs in which a significant change occurs between the sizes of corresponding blobs. As can be seen from this equation, in a perfect match, the blob size would be unchanged.

(6) $$Cost\left(G_{i}\right)=\underset{b\in P_{i}}{\sum }\frac{A\left(u\right)-\underset{v\in P_{i}}{\sum }A\left(v\right)}{\max\left\{A\left(u\right),\underset{v\in P_{i}}{\sum }A\left(v\right)\right\}}$$

(7) v(u)=1ifN≥k* logA,v(u)=0,otherwise

In the parasite level, our approach takes a different strategy than that proposed in [40] to be able to address issues occurring due to the closeness of parasites in the scene. The parasites level processing begins by assuming the association of exactly one parasite with each blob in the first frame of the video. Next, we iterate through the video and associate the parasites with their respective blobs in each frame. Specifically, if a parasite is associated with a blob in frame *i-1*, then it is also associated with all neighbors of that blob in frame *i*. Recall that the neighbors of each blob are given by the blob bipartite graphs computed at the blobs level. At this point, we estimate the number and location of parasites occurring within groups, where the results from the segmentation layer may not be highly accurate, based on a notion we call the *volatility factor *and denote by *v(u) *for a blob *u*. The volatility factor *v(u) *is determined based on the fraction of frames in which the blob *u *participates in splits or merges (See Eq.(7)). In this equation, *A *denotes the area of the blob (in pixels), *k *is a coefficient, and *N *defines the threshold number of frames. If a blob participates in splits/merges in large number of frames (exceeding a number *N*), then it is deemed to be volatile. Our hypothesis is that a blob having a high volatility factor is more likely to include erroneously segmented parasites than the one which has a low volatility factor. Operationally, to use this idea, we divide the blobs into three groups: small, medium, and large. Following Eq.(7), for small blobs, a split in approximately 20% of the frames was empirically determined to be the threshold for volatility. Similarly, for medium blobs, a split in 6% or more of the frames was used as the volatility threshold. Finally, for large blobs, a split in 1% of the frames was used as the threshold. Once the volatile blobs are identified, the tracker re-analyzes them starting with the first frame. To estimate the number and position of parasites in each blob, corresponding blobs from successive frames are overlaid and the underlying intensity values are superimposed to create a heat-map representing the possible locations of parasites. The bounding boxes of the blobs are then weighted and ranked based on the size of the box and the frequency of occurrence: a high scoring intensity box is expected to be close in size to the average bounding box of a single parasite. Excessively large intensity boxes are likely to contain multiple parasites, and very small intensity boxes are likely due to noise. The highest scoring intensity boxes are then considered in various combinations. For small blobs, combinations of two intensity boxes are considered, for medium blobs the combinations include up to three intensity boxes. Finally for large blobs, combinations of up to four intensity boxes are analyzed. These combinations are ranked to favor: (1) configurations where the participating intensity boxes are reasonably spread out (minimal overlap), (2) configurations where the total area of the combined intensity boxes is close to the area of the original parasite, (3) configurations where the bounding box of the combined intensity boxes roughly matches the dimensions of the bounding box of the original volatile blob. The highest ranked combination represents the most likely dimensions and locations of the new parasites. Figure 3(c) shows an example of a group of 4 parasites that was erroneously segmented as a single region in the initial frame of the video. For this case, ranking the combination of intensity boxes allowed the recovery of 1 of the 4 parasites.

### Quantitative description of phenotypes

In Table 1 we list the descriptors that capture the size, shape, motion (speed of movement), and appearance (intensity, color, and texture) of the parasites at every frame. These descriptors constitute the time-series representation of phenotypes used hereafter. Five classes of descriptors are used: size, shape, movement, texture, and color. Size descriptors measure the size of a parasite by the total number of pixels of the body of the parasite and the area change of the parasite between two consecutive time steps. The shape descriptor is calculated by dividing the length between two end points of the skeleton of the parasite by the skeleton length so that the ratio indicates how curved the parasite's body is. The movement of the parasite is measured by the pixel difference between two consecutive time steps: large changes in parasite shape or position lead to large difference value. Finally, color and texture descriptors capture the parasite appearance.

**Table 1:** Quantitative phenotype descriptors and their descriptions

Descriptor name	Formula	Description
**Size**		

Area	See description	The total number of pixels identified during segmentation.
Change in area	Area(*t*) - Area(*t*-1)	The area of the parasite in the current frame at time *t *minus the area of the parasite in the previous frame (time *t*-1).

**Shape**		

End point length/Skeleton length	See description	Ratio of the Euclidean length of the shortest line between the two endpoints of the skeleton to the length of the skeleton. The skeleton is created by thinning the segmented region until it is represented by a line corresponding to the curve of the body. Branching of the skeleton is handled by iteratively applying the MATLAB spur operator that identifies and removes isolated edge points until only two edges remain [47].

**Movement**		

Image difference	Image(*t*-1) - Image(*t*)	The number of pixels that moved from time *t*-1 to *t *of the parasite.
Perimeter (also for description of size)	See description	The number of pixels representing the boundary of the segmented region.
Axis ratio (also for shape description)	MinorAxisLength/MajorAxisLength	Ratio of the minor axis length to the major axis length. The major and minor axes are computed for an ellipse with the same normalized second central moments as the region.

**Texture**		

Entropy	-*sum*(*p*.*log_2_*p*)	Statistical measure of randomness related to the texture of an image where *p *contains the grayscale histogram.
Contrast	Σ|*i*-*j*|^2^*p*(*i*,*j*)	The intensity contrast between a pixel and its neighbors throughout the region.
Correlation	$\underset{i,j}{\sum }\frac{\left(i-\mu i\right)\left(j-\mu j\right)p\left(i,j\right)}{\sigma_{i}\sigma_{j}}$	The intensity correlation between a pixel and its neighbors.
Energy	$\underset{i,j}{\sum }{p\left(i,j\right)}^{2}$	The sum of the squared elements in the GLCM (gray-level co-occurrence matrix). The GLCM measures how often two intensities occur side by side.
Homogeneity	$\underset{i,j}{\sum }\frac{p\left(i,j\right)}{1+|i-j|}$	Measures the closeness of the distribution of the elements in the GLCM to the GLCM diagonal.

**Color**		

Average grayscale	See description	The mean intensity value and standard deviation found in the region.
Average red		
Average green		
Average blue		

Grayscale histogram	See description	A histogram with bins 0-255 representing the count of each intensity value present in the region.
Red histogram		
Green histogram		
Blue histogram		

### Time series analysis of phenotypes

In analyzing the phenotypic responses of individual parasites, our goal is to identify groups of similar phenotypic patterns. Conceptually, this problem requires clustering the phenotypes over time. However, when the time dimension is involved, the clustering problem becomes harder because each data point is not an individual instance but a sequence of data (collected over time). This implies that we are dealing with very high-dimensional data. Furthermore, given that our solution needs to work in high-throughput settings over large data sets, efficiency becomes a paramount consideration. Representing a time series symbolically constitutes one of the well known ways of complexity reduction. This approach, called SAX, has also been shown to improve clustering due to the smoothing effect of dimensionality reduction [41]. We briefly review the SAX approach (Section "Symbolic representation of time series (SAX)"). Then we show why the idea of representing a time-series with a fixed number of piece-wise linear segments as done in SAX is not directly applicable to our situation and propose a data-driven solution (Section "Automatic determination of piecewise aggregate approximation"). We discuss different distance measures for comparing the time-series generated in our problem (Section "Definition of a similarity measure between time-series"). The results from the aforementioned sections allow us to formulate the clustering techniques we use in this work to quantitatively analyze and differentiate the phenotypic response of parasites (Section "Clustering time series representation of phenotypes"). Finally, given clusters of similar phenotypic responses, we consider the questions of finding representative phenotypic models (Section "Identifying representative time series").

#### Symbolic representation of time series (SAX)

**Figure 4:**
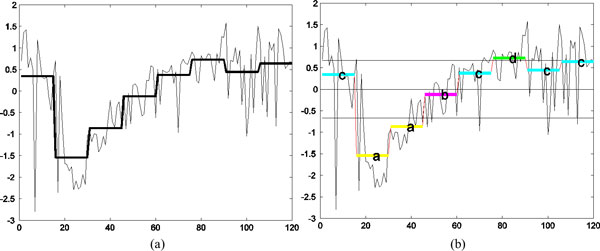
**(a) Original HTS data showing the effect of the drug Imipramine in terms of "Area" for a parasite and its PAA representation, (b) the symbolic representation.** The sequence of length 120 (*n *= 120) is reduced to 8 dimensions (*w *= 8) and is represented by the four SAX symbols (*a *= 4). X-axis denotes the frame number. The parasite body size (in pixels) is shown on the Y-axis.

SAX [41] represents a time series of arbitrary length *n *in a string of arbitrary length *w *(*w *<*n*, typically *w *<<*n*). This method consists of two steps. The first step involves a normalization of the original data to *N*(0,1), and then a transformation of the normalized data into a piecewise aggregate approximation (PAA) is performed. In the second step, the PAA is converted into a discrete string. Figure 4 shows an example of how a given time series of length *n *is represented in a *w*-dimensional space using the above steps. Each segment in the PAA indicates the average of the data points (PAA coefficient) along the segment. In this example, the length of the original data was 120 and the sequence was reduced to length 8. In the second step, a discrete representation is obtained based on producing symbols with equal probability. Given the normalized time series with Gaussian distribution from the first step, breakpoints are determined to divide the Gaussian curve into *a *equal-sized areas. These breakpoints are determined by looking up in a statistical table. Table 2 shows the breakpoints for values from 3 to 10. Once the breakpoints are obtained, a time series is discretized such that all PAA coefficients below the smallest breakpoint are mapped to the symbol "a", all coefficients greater than or equal to the smallest breakpoint and less than the second smallest breakpoint are mapped to the symbol "b", and so on. The three horizontal lines in Figure 4(b) represent the three breakpoints that produce four equal-sized areas under the Gaussian curve.

**Table 2:** A lookup table that contains the breakpoints

β	*a*							
	
	3	4	5	6	7	8	9	10
β_1_	-0.43	-0.67	-0.84	-0.97	-1.07	-1.15	-1.22	-1.28
β_2_	0.43	0	-0.25	-0.43	-0.57	-0.67	-0.76	-0.84
β_3_	−	0.67	0.25	0	-0.18	-0.32	-0.43	-0.52
β_4_	−	−	0.84	0.43	0.18	0	-0.14	-0.25
β_5_	−	−	−	0.97	0.57	0.32	0.14	0
β_6_	−	−	−	−	0.97	0.67	0.43	0.25
β_7_	−	−	−	−	−	1.15	0.76	0.52
β_8_	−	−	−	−	−	−	1.22	0.84
β_9_	−	−	−	−	−	−	−	1.28

#### Automatic determination of piecewise aggregate approximation

Directly applying SAX to large and varied data common to HTS, requires properly estimating the two control parameters *w *and *a*. Through extensive experiments, we found that values of the alphabet size *a *= 5 or *a *= 6 led to representations that allowed for good discrimination without being overly influenced by noise. A similar observation was also reported in [41]. However, we could not find any strategy that could consistently estimate a good value of *w *(the number of segments) *a priori*. We therefore propose a strategy to determine *w *in a data-dependent manner based on a method that was originally proposed for shape representation using optimal polygonal approximation of digital points [42]. This method uses the *L*_1 _norm to find the set of longest line segments, which fit the data with the minimum sum of absolute errors along each of the line segments. Given *n *data points and indices *i *and *j *such that *i = *1*,...,n-*2 and *j = i+*2*,...,n*, an objective function is defined as:

(8) $$F_{j}=L_{j}-E_{j},$$

where

(9) $$L_{j}=\sqrt{{\left(x_{j}-x_{i}\right)}^{2}+{\left(y_{j}-y_{i}\right)}^{2}}$$

and

(10) $$E_{j}=\left(\sum_{k=i+1}^{j-1}|\left(y_{j}-y_{i}\right)x_{k}-\left(x_{j}-x_{i}\right)y_{k}-x_{i}y_{j}+x_{j}y_{i}|\right)∕\sqrt{{\left(x_{j}-x_{i}\right)}^{2}+{\left(y_{j}-y_{i}\right)}^{2}},$$

**Figure 5:**
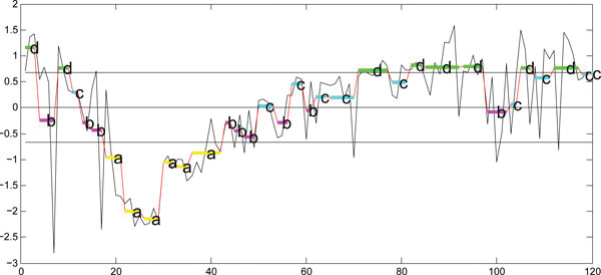
**Optimal segmentation and symbolic representation of the time-series from Figure 4(a).** The frame number is shown on the X-axis and the parasite body size (in pixels) is shown on the Y-axis.

The goal of the objective function is to maximize the length of a line segment *L_j _*and to minimize the error *E_j _*simultaneously. Algorithm 1 is the pseudo code that finds a set of break-points *P*(*x_j_*,*y_j_*) which produces optimal line segments from a given time series. Initially, *i *= 1 and *j *starts from *i*+2. If *F_j-1 _*≤ *F_j_*, then the value of *k *is incremented. Otherwise, *j-*1 becomes a breakpoint and a new segment is started with the two end points, *i *= *j-*1 and *j *= *i *+ 2. Since a time series is not a polygon but a curve along time dimension, we modify the original algorithm so that the start point of the new segment occurs after the end point of the previous segment (line 20 in Algorithm 1). Figure 5 shows the optimal segments and symbolic representation of the time-series from Figure 4. In it the number of segments can be seen to have increased from 8 to 32 and the length of each segment is determined solely by the data it represents.

Algorithm 1. *OptimalSegmentation*

1. *i *= 1

2. breakPoints ←{}

3. while *i *<*n*

4.    *j *= *i *+ 1

5.    if *j *== *n*

6.       break;

7.    end if

8.    compute *F_j_*

9.    while *j *<*n*

10.       *j *= *j *+ 1

11.       compute $F_{j}^{\prime }$

12.       if $F_{j}\leq {F_{j}}^{\prime }$

13.          $F_{j}\leftarrow {F_{j}}^{\prime }$

14.       else

**15.          *j*←*j***-1

16.          breakPoints ← breakPoints ∪*j*

17.       break;

18.    end if

19.  end while

20.  *i*=*j*

21. end while

Algorithm 2. *ExpandCluster*

**1. seeds ← getNeighbors(*D*, *p*, *ε***)

2. if size(result) >*MinPts*

3.    *p*.clusterId ← *none*

4.    return *False*;

5. else

6.    update *p*.clusterId

7.      seeds ← delete(seeds, *p*)

8.    while ~isempty(seeds)

9.       * currentP*←getFirstSeed(seeds)

10.       result ←getNeighbors(*D*, *currentP*, *ε*)

11.       if size(result) >= *MinPts*

12.        for *i *from 1 to size(result)

13.           *q←*get(result, *i*)

14.           if *q*.clusterId is unclassified or noise

15.              if *q*.clusterId is unclassified

16.                 seeds ←append(seeds, *q*)

17.              end if

18.              update *q*.clusterId

19.           end if

20.        end for

21.       end if

22.       seeds ←delete(seeds, *currentP*)

23.    end while

24.    return *True*

25. end if

#### Definition of a similarity measure between time-series

Given two time series *Q *and *C *and their symbolic representations $\hat{Q}$ and  , the distance between the two corresponding strings in SAX is given by Eq.(11):

(11) $$MINDIST\left(\hat{Q},Ĉ\right)=\sqrt{\frac{n}{w}}\sqrt{\sum_{i=1}^{w}{\left(dist\left({\hat{q}}_{i},{ĉ}_{i}\right)\right)}^{2}}$$

**Figure 6:**
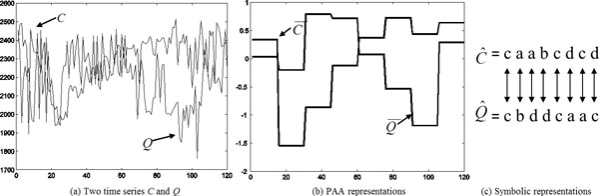
**(a) Two original time series *C *and *Q *(b) PAA representations of the two original sequences using the uniform segmentation (c) The symbolic representations of the two PAAs from (b).** X-axis denotes the frame number and the Y-axis denotes the body size (in pixels).

The *dist*() function in Eq.(11) is implemented by SAX using a lookup table defined in Table 3. For a given alphabet size *a*, the lookup table needs to be calculated only once. Figure 6 illustrates how two time series can be represented using symbols and how their similarity can be measured using SAX by matching the corresponding symbols. However, this method cannot be applied to our problem, since a data-driven PAA representation can map two time-series to symbolic representations of different lengths.

**Table 3:** A lookup table used by the MINDIST function, (*a *= 4)

	a	b	c	d
**a**	0	0	0.67	1.34
**b**	0	0	0	0.67
**c**	0.67	0	0	0
**d**	1.34	0.67	0	0

For comparing time-series as represented by symbolic representations of varying lengths, we investigate the following two distance measures:

*• Levenshtein distance measure *(LD) [43]: Given a source string *s *and a target string *t*, the LD between the two strings is defined as the number of deletions, insertions, or substitutions required to transform *s *into *t*. If *m *is the length of *s *and *n *the length of *t*, then computing LD requires filling a (*m*+1)×(*n*+1) distance matrix *D *with a distance between each pair of characters of the two strings. Initially, the first row of the matrix is set to 0,...,*m *and the first column of the matrix is set to 0,...,*n*. Next, for each cell *d*[*i*,*j*] in *D*, a value is computed as shown in Eq.(12). The final distance between the strings is given by *d*[*m*,*n*].

(12) d[i,j]= min{d[i-1,j],d[i,j-1]+1,d[i-1,j-1]+ cost},

where $i=0,...,m,j=0,...,n,\text{ and}\cos t=\left\{\begin{array}{cc} if\;s\left[i\right]==t\left[j\right], & 0\\ otherwise, & 1 \end{array}\right)$.

*• Dynamic time warping *(DTW): is an algorithm that finds an optimal alignment between two sequences. The warping path is found using dynamic programming using the following recurrence:

(13) r[i,j]=d[i,j]+ min{r[i-1,j],r[i,j-1],r[i-1,j-1]},

where *d*[*i*,*j*] is the distance in the current cell and *r*[*i*,*j*] is the cumulative distance.

#### Clustering time series representation of phenotypes

We investigate two different clustering methods for clustering the time-series based description of phenotypes: *agglomerative hierarchical clustering *and *DBSCAN*. Agglomerative clustering is a well known method for grouping biological data. DBSCAN [44] is a density-based clustering method and was originally developed for large spatial databases with noise. This method finds clusters with arbitrary shape, that is, it is not restricted to spherical clusters.

In DBSCAN, given an object *p *(time series in our case), an *ε*-*neighborhood *is used to denote other objects (time series) that lie within a radius *ε *of *d*. An object *p *is called a *core object *if it has a minimum number of objects, *MinPts*, in its *ε*-neighborhood. Given a set of objects, *p *is said to be *directly density-reachable *from a core object *q*, if *p *is within the *ε*-neighbor of *q*. Analogously, an object *p *is *density-reachable *from object *q *with respect to *ε *and *MinPts *if there is a chain of objects *p*_1_,..., *p_n_*, *p*_1 _= *q*, *p_n _*= *p *such that *p_i_*_+1 _is directly density-reachable from *p_i_*. Finally, an object *p *is density-connected to an object *q *with respect to *ε *and *MinPts *if there is an object *o *such that both *p *and *q *are density-reachable from *o *with respect to *ε *and *MinPts*.

Our use of DBSCAN starts with an arbitrary time series *p*, and retrieves all density-reachable time-series from *p *with respect to *ε *and *MinPts*. To estimate these parameters we analyzed the results from agglomerative clustering. The y-axis of the dendrogram is the distance between sequences. The linkages visually show which objects are closely located and helps users approximate the distances among objects. Therefore, in this paper, *ε *was set to the distance of two farthest objects within the cluster from the dendrogram. For the variable *MinPts*, we used the value of 2 or 3 not only to avoid singleton clusters but also to identify phenotypes that are distant from other phenotypes. In other words, if singleton is found, it means that the phenotype is truly distinct. If *p *happens to be a core object, then a cluster is generated. Algorithm 2 shows the pseudo code that checks whether *p *is a core object and expands a cluster if so. Initially, all objects are unclassified. For every unclassified object, *ExpandCluster *is called to see if the object is eligible to be a core object (line 2). If the object is indeed a core object, then all the neighboring objects within the radius *ε *are inserted into a queue (as they are directly density-reachable). For each object in the seeds any unclassified object in its *ε*-neighbors is inserted into the seed queue and is classified by being assigned a clusterId (line 18). By doing so, a cluster is formed by a set of density-connected objects that is maximally density-reachable. Every object not contained in any cluster is treated as noise.

**Figure 7:**
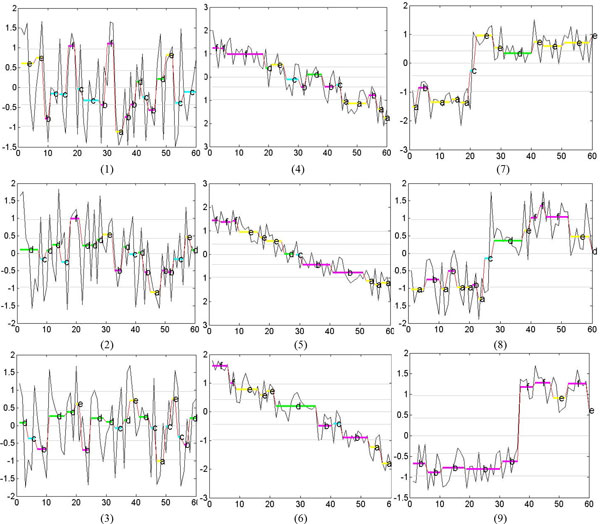
**Optimal segmentation and symbolic representation of the sequences from the UCI database with *a *= 6.** The X-axis denotes the time step and the sequence value is shown on the Y-axis.

**Figure 8:**
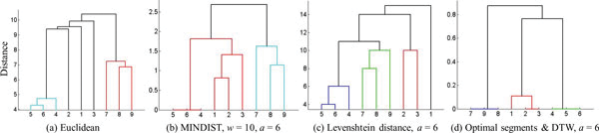
**Dendrograms constructed by the agglomerative hierarchical clustering for the UCI dataset.** The X-axis denotes the sequence number and the Y-axis denotes values obtained using various distance measures.

We use two data sets to investigate the applicability of hierarchical clustering and DBSCAN. The first data set is the synthetic control chart time series from the UCI machine learning repository. Because the class label of every sequence is known for this set, we use it for studying the validity of the resulting clusters. Three sequences of length 60 are selected from each of the three classes; normal, decreasing, and upward shift. Figure 7 shows these time series along with the optimally determined segments and the symbolic representations for each sequence. For the UCI data set, the dendrograms in Figure 8 show the clusters found by the agglomerative hierarchical clustering using four distance functions; Euclidean, MINDIST, Levenshtein distance, and DTW. The original time series were used for Euclidean distance and the symbolic representations were used for the other distance functions. As the Figure 8(a) shows, the three normal trends were not properly identified when Euclidean distance was used. For the original SAX method, the two control parameters were exhaustively explored and all combinations of alphabet size and segment length were tried (*a *= [3-8], *w *= [5,10,15,20,30]). Among the 30 combinations, the combination of *w *= 10 and *a *= 6 (Figure 8(b)) provided the best clustering with respect to the ground truth. For Levenshtein distance (Figure 8(c)) and DTW (Figure 8(d)), the automatically determined optimal segments were used. Calculation of DTW was also employed to compute distance between two strings. DBSCAN also found the cluster membership of each sequence, cluster1 = (1, 2, 3), cluster2 = (4, 5, 6) and cluster3 = (7, 8, 9).

**Figure 9:**
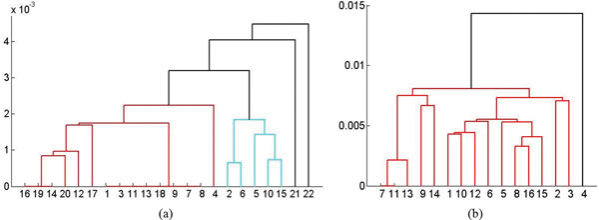
**Dendrograms constructed by agglomerative hierarchical clustering using values of the image difference descriptor: (a) Control group (b) Phenotypes measured on the 7^th ^day after exposed to drug Imipramine.** The X-axis: denotes the sequence number and the Y-axis denotes distance.

The second data used by us is a control group. For our experiment, we use the image difference descriptor and study 22 parasites. This descriptor corresponds to the motion exhibited by a parasite; greater the motion, larger the image difference. Figure 9(a) shows the dendrogram constructed by the agglomerative hierarchical clustering using this descriptor. The clusters found by DBSCAN using DTW were cluster-1 = (4, 12, 14, 16, 17, 19, 20), cluster-2 = (1, 3, 9, 11, 13, 18), cluster-3 = (7, 8), cluster-4 = (2, 5, 6, 10, 15), and noise = (21, 22) with *ε*= 0.0031 and *MinPts *= 3 for this data set. In Figure 9(a), cluster-1 and cluster-2 are linked by the same color (red) but DBSCAN separated them with the two parameter setting (*ε*= 0.0031 and *MinPts *= 3).

For this data set, no significant difference was found in the results from DBSCAN and agglomerative hierarchical clustering, indicating the validity of the clustering results of both methods. A manual inspection of the data confirmed this conclusion and the clusters that were obtained.

The third data set captures the effect of the drug Imipramine after parasites had been exposed to it for seven days. DBSCAN found two clusters for this data set, cluster-1 = (1, 7, 9, 11, 13, 14) and cluster-2 = (2, 3, 4, 5, 6, 8, 10, 11, 12, 15, 16) with *ε*= 0.03 and *MinPts *= 3. Clusters that are similar to the two clusters found by DBSCAN can be found by cutting off the black and the topmost red links in Figure 9(b). It confirms that there was no significant difference in clusters found by the two different clustering methods, DBSCAN and agglomerative hierarchical clustering.

#### Identifying representative time series

Finding a representative time series for a given cluster requires identifying one of the constituent time series which best characterizes the phenotypic diversity of the cluster. Different principles can be used to identify such a representative. In this paper, we propose two methods that approach this question from different perspectives. In the first method, we define the representative to be a time series that has the minimum sum-of-distances (MSD) with all the other time series in that cluster. We use DTW defined over the symbolic time-series representations to determine the representative.

The second method used by us is based on the notion of a low dimensional vector called sketch, which has been used for discovering approximately repeating subsequence [45]. The sketch vector $\vec{S}$ of length *k *for a subsequence vector $\vec{t}$of length *n *is defined as:

(14) $$\vec{S}\left[i\right]=\sum_{j=1}^{n}t\left[j\right]\cdot v_{i}\left[j\right]$$

In Eq.(13), *i*=1,..,*k *and ${\vec{v}}_{i}$ is a random vector. Each element of ${\vec{v}}_{i}$ is an independent random variable with normal distribution *N*(0,1) and the magnitude of the vector is normalized to 1. For example, $\vec{S}$ of length 2 for $\vec{t}=\left(3,1,2,4\right)$ is computed as following: first, two random vectors are chosen and then normalized, ${\vec{v}}_{1}={\left(-0.6895,0.1717,-0.2970,-0.8008\right)}^{T}$ and ${\vec{v}}_{2}={\left(0.\text{47}0\text{8},-0.\text{2446},-0.0\text{45}0,0.\text{2179}\right)}^{T}$. Next, the sketch vector is computed using Eq. (13). In this example, $\vec{S}=\left(\vec{t}\cdot {\vec{v}}_{1},\vec{t}\cdot {\vec{v}}_{2}\right)=\;\left(-\text{5}.\text{694}0,\text{ 1}.\text{9494}\right)$. In our method, all the time series in a cluster are transformed to sketches of length 30. Once a sketch pool is obtained for the cluster, the *L*_2 _norm is applied to identify a sketch with the least sum of distances to all other sketches and the time series corresponding to this sketch is declared as the cluster representative.

**Figure 10:**
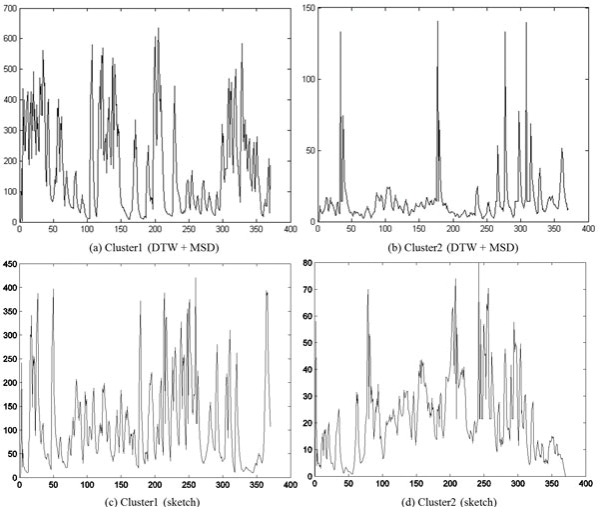
**Representative time series of the two clusters.** Note that the magnitude of (a) and (c) are greater than that of (b) and (d). The frame number is depicted on the X-axis while the Y-axis denotes the change in area.

**Figure 11:**
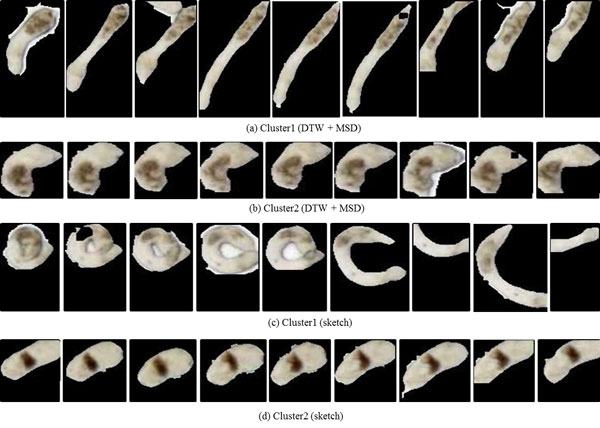
**Shape change in the parasites corresponding to the representative time series for each cluster.** The snapshots depict parasites at the first frame and at every 45^th ^frame thereafter. As can be seen, based on the rate of shape change, the parasites in (a) and (c) are more active than those in (b) and (d).

**Figure 12:**
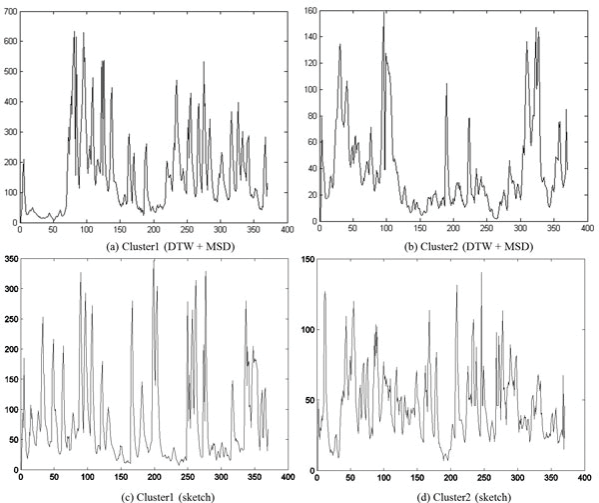
**Representative time series of the two clusters.** The data was collected on the seventh day after exposure to the drug Imipramine. Note that the magnitude of (a) is greater than that of (b) and the magnitude of (c) is also greater than that of (d). In this figure the frame number is depicted on the X-axis and the change in the size of the parasites is shown on the Y-axis.

**Figure 13:**
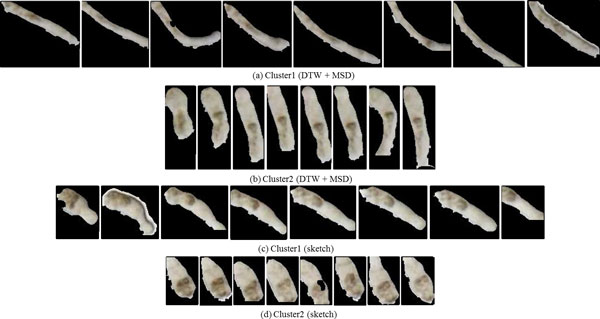
**Snapshots depicting the shape change in the parasites corresponding to the representative time series for each cluster from Figure 12 at the first frame and every 45^th ^frame thereafter.**

In Figure 10 we show the representative time series identified by DTW and sketching for the two clusters obtained by cutting the links off around 0.003 in Figure 9(a). As the reader may note, the movement of the representative parasite in cluster1 is greater than that of the representative parasite from cluster 2, with both the methods. This behavior is consistent with manual observations. In Figure 11, we show the shape change of the representative parasites from each of the two clusters (every 45^th ^frame in the observation period is depicted). Since the movement occurred over the same time-duration, the reader can see greater motion exhibited by parasites from the first cluster. Since the parasite identified using the sketch shows greater mobility than the one obtained using DTW+MSD, we postulate that sketching may be a better approach for finding representative time series. Figure 12 shows the representative time series identified by DTW and sketching for the two clusters obtained on the 7^th ^day after exposed to the drug Imipramine. Figure 13 shows the shape change of the representative parasites from each of the two clusters.

### Experiments

**Figure 14:**
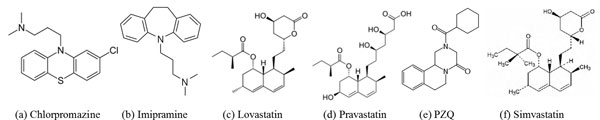
**The structures of the six compounds used in the experiments.**

In the following, we present a number of experiments and case studies to validate the proposed method and apply it to analyze data from phenotypic screens. The data used in this experiment was generated by screening six compounds which are shown in Figure 14. These compounds were chosen from published whole-organism screening activities to reposition and potentially fast-track known drugs as therapy for schistosomiasis. As visually interpreted using the constrained nomenclature from [20], these compounds elicited consistent, striking and disparate responses from schistosomula. These responses included: parasite hyper-motility (induced by the tricyclic neuroactive compounds chlorpromazine and imipramine), darkening and decreased motility leading to death (induced by the anti-hypercholesterolemia drugs: lovastatin, pravastatin and simvastatin). Finally, being the current therapy for schistosomiasis, PZQ, was also chosen. This compound generated a variety of dose-dependent responses not seen with the other compounds that included shrinking and degeneration combined with hyper-motility. The phenotypes analyzed by us in this paper were recorded 7 days after the exposure of the parasites to the drug.

We begin by presenting results that quantify the accuracy of the image segmentation and tracking. Next, experiments related to time series clustering and the identification of representative time series are presented. Included in this section are results from a case study which was conducted to compare the phenotypic response within two groups of parasites. The control constituted the first group while the second group was exposed to the current gold standard drug PZQ. An important result from this study was that the effect of PZQ could be stratified in terms of three distinct phenotype clusters. Following this, in the section "Cluster identification using phenotypes from control and multiple compounds", the ability of the proposed method to automatically segregate phenotypes arising from the effect of different compounds is analyzed. A key goal of this analysis was to find the best combination of the alphabet size, the segment length and the distance function for use in subsequent experiments. Results of using these parameters for classifying the phenotypes elicited by the six compounds are presented in Section "Clustering of phenotypes elicited from all compounds". These results constitute proof of concept for the proposed method. Finally, in the section "Identification of representative time series" we present a case study that involves determining the representative phenotype models for the controls as well as for parasites exposed to different compounds.

### Analysis of the accuracy of image segmentation and tracking

To determine the effectiveness of the segmentation and tracking, we manually counted the number of parasites in five videos (See Table 4). The manual counts represented the "ground truth" for this sample. Next, the ground truth was compared to the number of parasites which were correctly segmented and the number of parasites which were correctly tracked across the video. The third column of Table 4 (Segmented Parasites) shows the number of correctly segmented parasites in the first frame of the corresponding video. The next column (Tracked Parasites) shows the number of parasites which were correctly tracked throughout the entire duration of the video. Overall, the tracker successfully tracked 224 out of 272 parasites (82.3%). What is notable about this statistic is that across all the videos only 206 out of 272 parasites (75.7%) were correctly segmented on the first frame. Thus, these results highlight the fact that tracking can lead to improved parasite segmentation. Specifically, for the given data set, the overall improvement in parasite segmentation was about 6.6%. Additionally, there was a 10.6% false positive rate, due to errors in tracking.

**Table 4:** Image segmentation and tracking accuracy of five groups

Compound	Total parasites	Segmented parasites	Tracked parasites	False positives	Segmentation accuracy	Tracked accuracy
Pravastatin	50	33	41	3	66%	82%
Simvastatin	67	58	62	5	87%	93%
Imipramine	53	43	39	4	81%	74%
PZQ	42	27	31	8	64%	74%
Control	60	45	51	9	75%	85%

### Data pre-processing and parameters employed in time-series analysis

The video data was collected and analyzed using the methods described in Sections "Parasite identification by image segmentation" and "Parasite tracking". As is well known, real-world data tends to be noisy. To reduce the noise, each value of the descriptor in the time series was replaced by the mean of neighboring values within a sliding window and then outliers were smoothed out by a density-based local outlier detection method [46]. If an object does not have minimum number of neighbors within certain distance, the object is considered as an outlier. In all the experiments, the alphabet size used by us for transformation of the time-series to a symbolic representation was either 5 or 6. The video recordings were also manually analyzed by experts using the protocol developed in [20] and qualitative descriptors were assigned to each video. These qualitative descriptors were used by us for independent validation of our results.

#### Case study: analysis of phenotypes of control vs PZQ

**Figure 15:**
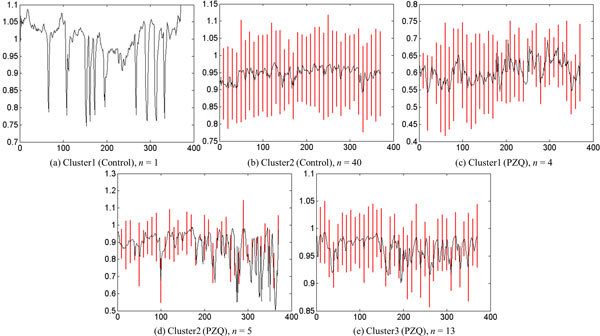
**Representative time series and standard deviation at every 10^th ^frame, *a *= 6.** (a) Cluster1 is singleton so its standard deviation is 0. It is therefore not shown in the plot. The cluster size is denoted by *n*. The X-axis denotes the frame number and the Y-axis denotes the ratio of the end point length to the skeleton length.

**Figure 16:**
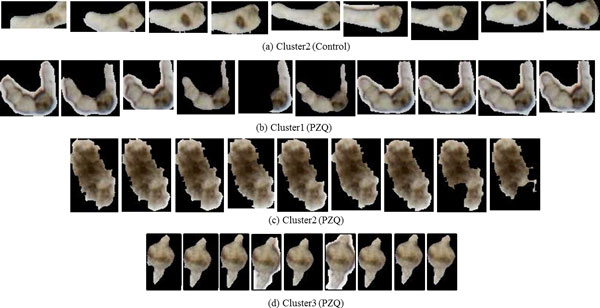
**Shapes of the representative parasites over time.** Starting from the first frame, the snapshots are taken every 45^th ^frame. (a) Representative of the dominant cluster in the control data. (b - d) The representative parasites from each of the three clusters found in the set that was exposed to PZQ. The reader may note the distinct differences of the phenotypic response of the parasites in each of the clusters.

In this case study, we analyzed the phenotypic differences exhibited by control parasites and those exposed to PZQ. Two clusters were identified for the control group. Cluster1 was a singleton (*n *= 1) and cluster 2 was the dominant cluster (*n *= 40). The standard deviations of cluster 2 (Figure 15(b)) at every 10^th ^frame were found to be nearly uniform. Figure 16 shows the shape change of the representative parasites of control group and the group exposed to PZQ. The reader may note that the shape descriptor for this data set not only gave us the shape information but also provided information regarding the frequency with which the parasite moved due to the fact that parasite is correlated with change in shape over time. The clustering result for the control group means that most parasites in this group had similar shape over time. On the other hand, the presence of three clusters amongst the parasites exposed to PZQ seemed to indicate that the effects of the drug were manifested differently in different parasites. This is shown through the snapshots of the three clusters (b, c, and d in Figure 13).

#### Cluster identification using phenotypes from control and multiple compounds

**Figure 17:**
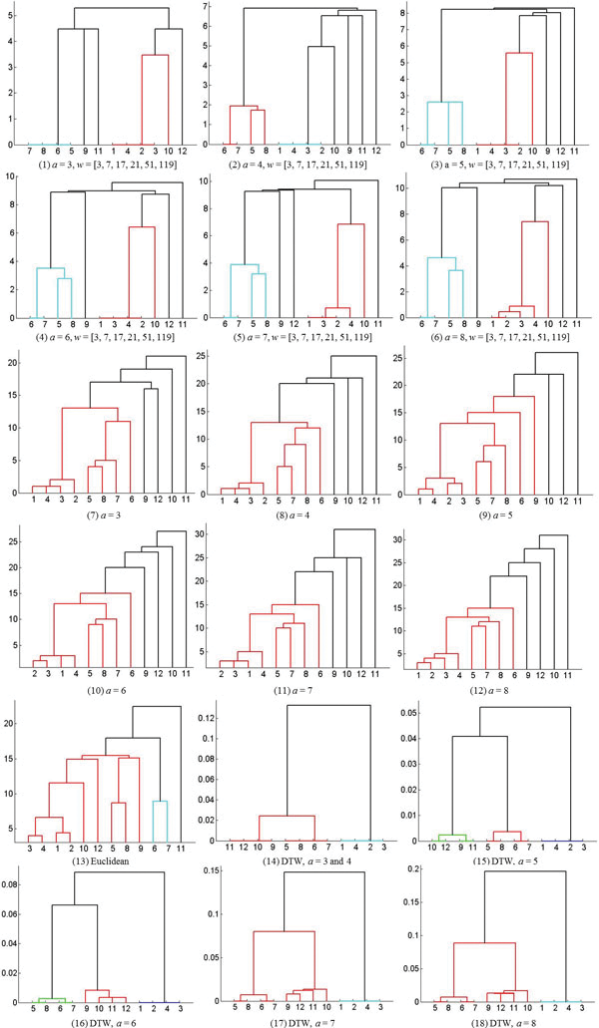
**Dendrograms constructed by various distance functions, alphabet sizes and segment lengths.** Control group = {1, 2, 3, 4}, PZQ = {5, 6, 7, 8}, Lovastatin = {9, 10, 11, 12}. Dendrograms constructed using SAX and MinDist with varying alphabet sizes and varying number of segments are shown in parts (1-6). For each case, the six segments (*w *= [3, 7, 17, 21, 51, 119]) resulted in the same clusters. In parts (7-12) dendrograms constructed by optimal segmentation and edit distance are shown. The dendrogram constructed using Euclidean distance is shown in (13). In parts (14-18) dendrograms constructed using the proposed method are depicted. In our method, different alphabet sizes did not lead to differences in clustering accuracy. In this figure the sequence number is shown on the X-axis and the distance values are shown on the Y-axis.

The goal of this experiment was to find the best combination of the alphabet size, the segment length and the distance function so as to separate the phenotypic response of parasites that were exposed to different compounds. We used the shape descriptor defined as the ratio of the end-point distance to the length of the skeleton. This descriptor is especially well suited to distinguish parasites having normal shape from those that are straight. Four samples were selected from the three groups: control, PZQ and Lovastatin. The sampling was made in the following way. For each group, all of the time series were optimally segmented and then symbolically represented using our proposed method. Given the strings, clusters were found by the agglomerative hierarchical clustering and then four samples were selected from each one of the clusters. Four distance functions were tried by the agglomerative hierarchical clustering: MinDist of the original SAX method, edit distance, Euclidean distance and DTW. The distance for every pair of the symbolic representations of time series sequences was computed by each of the three distance functions and then the clusters of those time series sequences were identified based on the distances. When Euclidean distance was used, the raw data were used instead of the symbolic representation. A data set was formed by the total 12 time series from the three groups; Control = {1, 2, 3, 4}, PZQ = {5, 6, 7, 8}, Lovastatin = {9, 10, 11, 12}. Note that the parasites from the control group were slim and long, while those treated with PZQ & Lovastatin had curved and oval shapes respectively. By doing so, the data set was clearly separated and the ability of the method to distinguish the phenotypes could be tested unambiguously. Three distance functions were tried to investigate the clustering accuracy. In Figure 17 we show the dendrograms (Figure 17(1)-(6)) constructed by SAX [41]. For the original SAX method, the 36 combinations by the six alphabet sizes (*a *= [3, 4, 5, 6, 7, 8]) and six segment lengths (w = [3, 7, 17, 21, 51, 119]) were tried. As the dendrograms show, the parasites from the control and PZQ groups were clearly identified but the parasites from the Lovastatin group were not clearly separated from the parasites from the control and PZQ groups. The various combinations of the six alphabet sizes and the six segment lengths did not make much difference in the clustering as observed in dendrograms in Figure 17.

Next, the Levenshtein distance was applied to the symbolic representations of optimally segmented time series. The parasites from the control group were found to be well separated but the other two groups were not properly distinguished (Figure 17(7)-(12)). The results using the Euclidean distance are shown in Figure 17(13). The final distance function we investigated was DTW. The six different alphabet sizes were tried and for all of them, the three clusters could be clearly identified. As the results show (Figure 17(14)-(18)), the alphabet size did not affect the clustering.

### Clustering of phenotypes elicited from all compounds

This experiment represented an extension of the one carried out in the previous section with the random choice of parasites. Here phenotypic responses of parasite exposed to each of the compounds were collected from video observations made on the 7^th ^day. Four parasites were randomly selected from each of the seven groups including the control group as follows: Control = {1 - 4}, Chlorpromazine = {5 - 8}, Imipramine = {9 - 12}, Lovastatin = {13 -16}, Pravastatin = {17 -20}, PZQ = {21 - 24}, and Simvastatin = {25 - 28}. Perceptually, the appearance of the parasites constituted the most significant phenotype. Consequently, in this experiment the average grayscale intensity was used as the descriptor. The agglomerative hierarchical clustering algorithm was employed using DTW distance. The results of the clustering along with the ground truth and a manual description of the parasite appearance are shown in Table 5. A very high accuracy of clustering was obtained (error rate of 0%) with respect to the ground truth.

**Table 5:** Clusters obtained for phenotypes elicited from all compounds

Compound	Cluster 1	Cluster 2	Manual phenotype assignment (based on parasite appearance)
Control		1, 2, 3, 4	Light
Chlorpromazine	5, 7		Dark
		6, 8	Light
Imipramine	9, 11, 12		Dark
		10	Light
Lovastatin	13, 14, 15, 16		Dark
Pravastatin		17, 18, 19, 20	Light
PZQ		21, 22, 23, 24	Light
Simvastatin	25, 26, 27, 28		Dark

### Identification of representative time series

**Figure 18:**
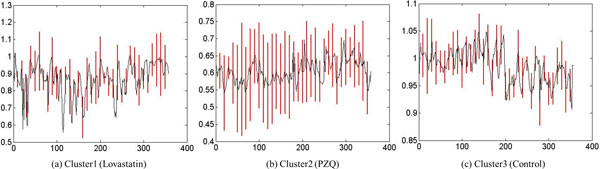
**Representative time series and standard deviation of each cluster, *a *= 4.** The frame number is shown on the X-axis. The Y-axis depicts the descriptor: End Point Length/Skeleton Length.

**Figure 19:**
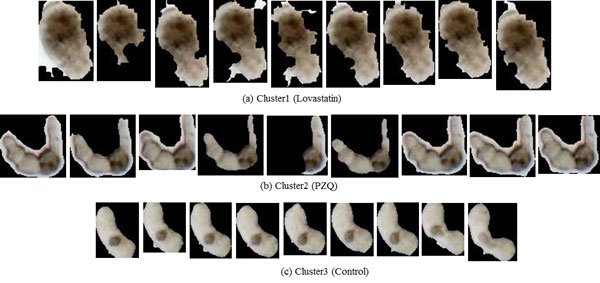
**Representative parasites for each of the three clusters.** The figure shows snapshots that were captured starting at the first frame and at every 45^th ^frame thereafter.

In this experiment, we further analyzed the data from Section "Cluster identification using phenotypes from control and multiple compounds", where a shape descriptor was used to cluster the phenotypes arising as a response to Lovastatin and PZQ in comparison to the control. The representative time series of each cluster (Figure 18) was found by sketching and DTW. The descriptor values for PZQ (Figure 18(b)) were amongst the smallest for the three drugs, indicating that the drug caused the parasite body to curl. The snapshots of the three parasites of the representative time series for the three groups are shown in Figure 19. In this figure, the clear difference in the shape change can be observed. Furthermore, DBSCAN was also clearly able to identify the membership of each time series with parameters *MinPts *= 2 and *ε *= 0.012; {1, 2, 3, 4}, {5, 6, 7, 8} and {9, 10, 11, 12}. This experiment along with its counterparts demonstrates that the proposed approach can be used for accurately grouping and quantifying phenotypic responses to different drugs. Furthermore, the method can also provide, automatically, the representative phenotype models present in the data.

## Conclusions

The research presented in this paper represents a significant breakthrough towards quantitative phenotypic drug screening against neglected diseases, such as schistosomiasis, where the effect of a drug on the target pathogen is manifested through a continuum of complex phenotypes. The proposed method lies at the interface of disciplines. From the algorithmic perspective, a key contribution of this work has been the adaptation and extension of techniques from time-series data analysis for representation and reasoning about phenotypes exhibited by schistosomula. An important result from this perspective has been the development of a rigorous approach to automatically quantify and characterize the phenotypic responses different parasites to a drug. Consequently, the proposed method can be crucial for development of high-throughput phenotypic screens where a much larger fraction of the chemical space can be explored during lead discovery. Another important result lies in the ability of the method to detect and represent variability in the response of different parasites when they were exposed to the same drug in identical environmental conditions. Recognizing such stratifications in the parasite population may be significant in more ways than one. Among others, detection of such variability can play a major role in driving exploration of the pathogen's biology and in understanding the development of resistance to drugs. Furthermore, the existence of such variability also underlines the need for developing new computational and statistical methods that can robustly analyze highly variable data from high-throughput phenotypic screens.

## Competing interests

The interpretation of the data, findings and conclusions contained within this paper were not influenced by personal, financial and non-financial relationships with the funders.

## Authors' contributions

RS formulated the problem and proposed the time-series analysis-based solution framework. The methods for image segmentation and image feature extraction were designed and implemented by AMD and RS. US and RS designed and implemented the tracking algorithm. The time-series analysis methods were designed and implemented by HL and RS. BMS, CRC, SC, and MA were involved in assay design. The video data was captured by BMS and CRC. DA was responsible for storage and management of the video data. The computational experiments were conducted and analyzed by HL, RS, AMD, and US. The paper was written by RS and HL. All authors read and approved the final manuscript.
